# Colorectal Cancer Incidence in Iran Based on Sex, Age, and Geographical Regions: A Study of 2014–2017 and Projected Rates to 2025

**DOI:** 10.34172/aim.2024.26

**Published:** 2024-04-01

**Authors:** Hani AziziKia, Azin Teymourzadeh, Hosein Kouchaki, Amin Nakhostin-Ansari, Pooya Jafari Doudaran, Izadmehr Ahmadinejad, Armin Hoveidaei, Gholamreza Roshandel

**Affiliations:** ^1^Student Research Committee, School of Medicine, Shahroud University of Medical Sciences, Shahroud, Iran; ^2^Imam Khomeini Hospital Complex, Tehran University of Medical Sciences, Tehran, Iran; ^3^Shiraz Institute for Cancer Research, School of Medicine, Shiraz University of Medical Sciences, Shiraz, Iran; ^4^Sports Medicine Research Center, Neuroscience Institute, Tehran University of Medical Sciences, Tehran, Iran; ^5^Faculty of Medicine, Qom University of Medical Sciences, Qom, Iran; ^6^Student Research Committee, Tehran University of Medical Sciences, Tehran, Iran; ^7^Student Research Committee, School of Allied Medical Sciences, Shahid Beheshti University of Medical Sciences, Tehran, Iran; ^8^Golestan University of Medical Sciences and Health Services, Gorgan, Iran

**Keywords:** Colorectal cancer, Incidence, Iran, Projection

## Abstract

**Background:**

While there has been extensive research on colorectal cancer (CRC) incidence and its associated factors in Iran, a significant gap exists in studies predicting its future trends. Our study aimed to thoroughly report CRC incidence across Iran from 2014 to 2017, by sex, age, and geographical regions, and provide a projection for 2025.

**Methods:**

This retrospective study utilized data from the Iranian National Population-based Cancer Registry (INPCR). Patients with the International Classification of Diseases for Oncology, 3^rd^ Edition (ICD-O-3) codes C18 to C21 were included. The age-standardized incidence rate (ASR), was calculated per 100000 individuals annually, and crude incidence rates were retrieved for various demographic groups and years.

**Results:**

Between 2014 and 2017, a total of 43580 new CRC cases (55.96% males) were registered. Men exhibited an ASR of 134.45, while women’s ASR was 94.85. The highest ASRs were observed in Tehran, Qom, and Ilam (18.99, 18.26, and 18.06, respectively). Incidence rates surpassed 20 after age 50 for both genders, reaching their peak within the 80–84 age group. Adenocarcinoma was the most frequent histological type of CRC in nearly all provinces. Case numbers and ASRs are projected to continuously rise until 2025, with a predominance of male cases.

**Conclusion:**

The anticipated increase in CRC incidence in Iran emphasizes the need for additional studies to better identify risk factors. Furthermore, implementing screening programs is recommended for individuals at a higher risk of CRC, including men, the elderly population, and those residing in regions with a notable prevalence of CRC.

## Introduction

 Colorectal cancer (CRC) refers to a group of tumors that develop in the colon and/or rectum. Globally, it ranks third among the most frequent cancers and is also the second leading cause of cancer-related mortalities.^1^ However, the CRC incidence rate exhibits remarkable disparities worldwide. Notably, Australia and New Zealand report the highest CRC rates, followed by Western Europe and North America.^2^ On the other hand, CRCs are less prevalent in regions like Africa and South-Central Asia.^2^ The inequality in the global incidence of CRC highlights the significance of regional-related factors in the development of this cancer, including genetic background and diverse lifestyle characteristics among different populations. Overall, several factors contribute to CRC development, including both modifiable and non-modifiable risk factors. Non-modifiable factors for CRC include male sex,^3^ advanced age,^4^ black ethnicity,^5^ comorbidities like IBD^6^ and diabetes mellitus,^7^ and having a positive history of CRCs in first-degree relatives.^6^ On the other hand, some of the modifiable lifestyle-related factors include obesity,^6^ low physical activity,^8^ regular alcohol consumption,^9^ smoking,^9^ and adopting unhealthy diets such as the red-meat-rich diet.^6^ Developed countries have dedicated significant efforts to managing the burden of CRC in the past decade, leading to either stabilization or reduction in disease rates within these regions.^10^ Conversely, the incidence of CRC is still rapidly growing in numerous low-income and middle-income countries.^11^

 Urbanization, an aging population, adoption of sedentary lifestyles, and Westernized diets have been on the rise at a faster rate among the Iranian population in recent years.^12^ This trend has led to a transition from infectious diseases to non-communicable ones. In this context, there has been an increasing trend in the number of individuals diagnosed with CRC in Iran.^13^ Currently, CRC is the third most common cancer in men and the second in women in Iran.^14^ Although CRC was the 7th leading cause of disability-adjusted life years (DALYs) among Iranian men and the 25th among Iranian women in 1990, it rose to the fourth rank for DALYs in both sexes by 2017.^14^ Furthermore, the mortality rate associated with CRCs has demonstrated a significant rise, escalating from 2.87 (2.4 – 3.5) in 1990 to 6.8 (6.0 – 7.1) in 2017.^15,16^ Understanding the various aspects of CRC in Iran, such as epidemiological characteristics (temporal shifts and geographic distributions), age- and sex-related trends of disease, and common subtypes, plays a pivotal role in effectively preventing and managing cancer within the country.

 This study used data on CRC incidence in Iran from the Iranian National Population-based Cancer Registry (INPCR). This database presents a distinct chance to gain insight into the landscape of CRC in Iran. We outlined the geographic distribution of CRC occurrences, analyzed the changes in its incidence rates between 2014 and 2017, and projected the anticipated CRC incidence up to 2025. Furthermore, we examined the changes in CRC incidence in Iran according to age, sex, and disease subtypes.

## Materials and Methods

###  Study Design and Population

 The present study was a retrospective investigation utilizing data from the INPCR dataset.^17^ The INPCR covers the entire population of Iran. Iran is one of the countries located in the Middle East, with 31 provinces. This country covers an area of 1.648 million square kilometers and hosts a population of near 80 million based on the last Iranian National Census in 2016. According to this report, 50.66% of this population were men and roughly half of them lived in rural areas. In addition, the mean age of the Iranian population was 31.1 years.

###  Organization of INPCR

 The national cancer registry system in Iran was initially established in 1999, by including pathologically confirmed cases of cancer. However, this initial system did not include significant data points, such as clinical findings and cancer-related mortality information. A more comprehensive program, known as the INPCR, was introduced by the Iranian Ministry of Health and Medical Education (MOHME) in 2014 in order to register Iranian patients diagnosed with cancer. The INPCR included all patients who were assigned an International Classification of Diseases for Oncology (ICD-O) code during the microscopic verification process, or those whose medical records and death certificates indicated cancer as the final diagnosis.^17^ This cancer registry system covers all 60 medical universities within the 31 provinces of Iran. Each medical university provides comprehensive health information about its designated coverage area. Three sources (pathology reports, clinical histories, and death certificates) are employed to gather data on cancer patients. Consequently, the collected data is integrated into the INPCR, resulting in a substantial national-level cancer database. In order to ensure data comparability, the INPCR established a national guideline for population-based cancer registries. This guideline is founded on the standard protocols and work plans recommended by international organizations like the International Agency for Research of Cancer (IARC) and the International Association of Cancer Registries (IACR).^17^ Following the guidelines, the INPCR recorded only cases of new primary tumors displaying malignant behavior. Moreover, regarding cases of metastasis or recurrent cancers, only the primary tumors were documented.^18^

###  Data Collection

 The cancer registries secretariat of the universities employed a blend of diverse data collection methods, including both electronic and paper-based approaches. However, they followed the same protocol in this context. Pathology reports from pathology centers, along with clinical and paraclinical data from hospitals, constituted the primary sources for data collection. Information was gathered from a total of 1540 distinct sources, encompassing 324 hospitals and 1216 pathology laboratories from 2014 to 2017. All these centers adhered to a uniform data entry protocol and minimized duplication by implementing an integrated system that utilized the patients’ national identification (ID) numbers. In addition to recording the national ID number, other demographic details including first name, last name, patronymic, age, and sex were also registered for data integration. For data entry, the initial revised third edition of the ICD-O (ICD-O-3) code is utilized, wherein CRC is coded as C18 to C21.^18-20^

###  Data Processing and Quality Control

 The quality control was initiated at university levels by internal consistency checking, including rechecking topography codes with morphology, age, and sex. Following the conclusion of data processing and quality control within each university cancer registry, the resultant data were annually submitted to the INPCR Secretariat. The INPCR Secretariat then carried out additional quality control evaluations, emphasizing data completeness and accuracy (based on morphology, age, and sex). Once the data had successfully passed the final INPCR quality control checks, they were deemed acceptable and utilized for the ultimate analysis.

###  Statistical Analysis

 At provincial and national levels, data were analyzed and reported as numbers, percentages, crude incidence rates, age-specific incidence rates, and age-standardized incidence rates (ASRs). The Canreg-5 software^21^ was used to calculate ASRs for the world standard population in 18 age categories of 5 years (0-4, 5-9..., 85 +). All rates were expressed as a percentage of a population of 100 000. ASRs were calculated using the Segi-Doll world population.^22^ The predicted numbers of cases and corresponding rates for CRC in 2020 and 2025 were calculated, based on fitting the INPCR data to the following time-linear age-period model developed by Dyba and Hakulinen:

 E(rate(i,t)) = αi + βi × t ^23^

## Results

**Figure 1:**
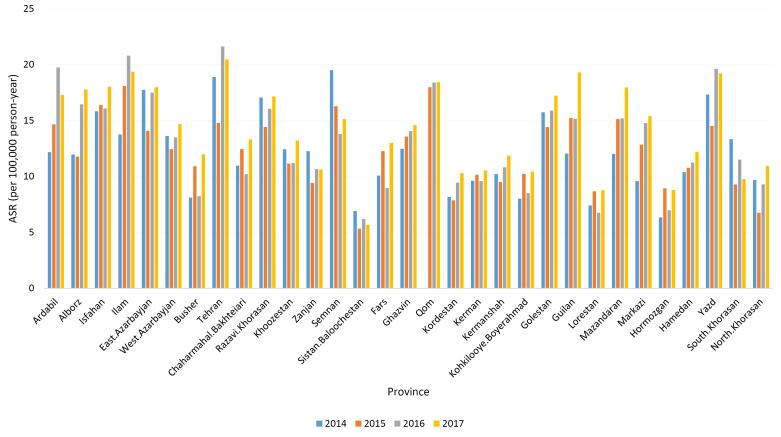
Age-Standardized Incidence Rate (Per 100 000 Person-Year) of Colorectal Cancers in Iran by Province, 2014–2017

 In this study, a total of 43 580 new cases of CRC (including 55.96% males) were recorded between the years 2014 and 2017. The overall ASR was 114.49, with 134.45 for males and 94.85 for females. Our findings suggested an increasing trend in the ASR of CRC among the whole population (from 13.80 in 2014 to 15.44 in 2017). Table 1 and Figure S1 display the number, crude incidence rate and ASR of CRC based on case registration for each year, from 2014 to 2017. As Table 1 reveals, both the crude incidence rate and ASR were higher in men during these four years. We also demonstrated the incidence of CRC (ASR) in all provinces of Iran, between the years 2014 and 2017. According to our findings, the ASRs of CRC in all areas exhibited an increasing trend from 2014 to 2017 (Table S1 and Figure 1). However, in 2015, there was a slight reduction in the crude incidence rate and ASR for CRC in approximately half of the areas (15 provinces out of 31), as shown in Figure 1. In 2014, Tehran, East Azarbaijan, and Yazd had the highest ASR, while in 2017, Tehran, Ilam, and Guilan reported the highest ASR.

**Table 1:** Number, Crude Rate, and Age-Standardized Incidence Rate (Per 100 000 Person-Year) of Colorectal Cancers in Iran by Year, 2014–2017

**Year**	**Total**			**Male**			**Female**		
	**Number**	**Crude rate**	**ASR**	**Number**	**Crude rate**	**ASR**	**Number**	**Crude rate**	**ASR**
2014	9867	12.85	13.80	5647	14.54	15.93	4220	11.12	11.73
2015	9817	12.43	13.13	5395	13.49	14.57	4422	11.34	11.73
2016	11404	14.27	14.93	6420	15.85	17.00	4984	12.64	12.93
2017	12492	15.44	15.96	6929	16.89	17.87	5563	13.95	14.12

ASR, age-standardized incidence rate.

**Figure 2:**
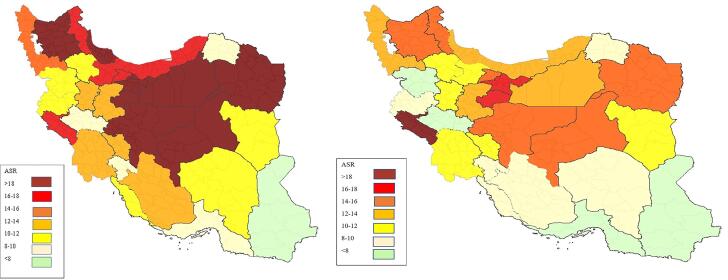
Age Standardized Incidence Rate of Colorectal Cancer in Different Provinces by Sex. (A) males, (B) females

**Figure 3:**
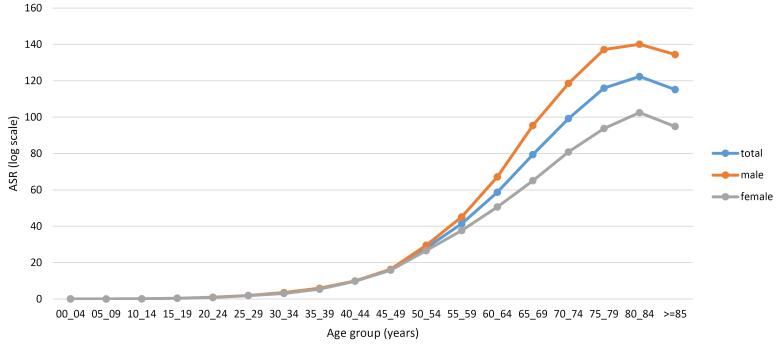
Age-Standardized Incidence Rate (Per 100 000 Person-Year) of Colorectal Cancers in Iran by Age Group and Sex, 2014–2017

**Figure 4:**
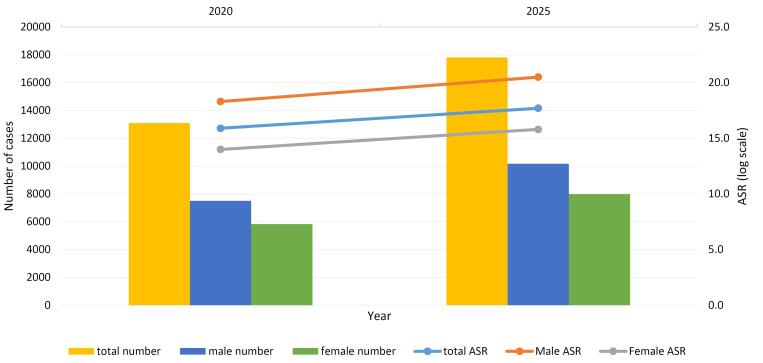
Prediction of Age-Standardized Incidence Rates (Per 100 000 Person-Year) of Colorectal Cancers in Iran, 2020 and 2025

 The number of CRC cases and ASRs by province of residence are shown in Table S2 (see Supplementary file 1). Among the 31 provinces of Iran, the highest ASRs were observed in Tehran, Qom, and Ilam, with rates of 18.99, 18.26, and 18.06, respectively. In contrast, the lowest ASRs were recorded in Sistan-Balouchestan (6.01), Hormozgan (7.80), and Lorestan (7.90). Figures 2 (A and B) depict the ASRs in different provinces for males and females, respectively. In addition, as illustrated in Figure 3, the ASRs exceeded 20 in both sexes once the individuals crossed the age threshold of 50 years. In this regard, the highest ASR was identified in the 80-84 age group, with 140.1 per 100 000 person-years and 102.48 per 100 000 person-years in males and females, respectively (Figure 4, Table S3).

 We also assessed the histopathological characteristics of CRC patients and have presented these findings in Table 2, categorized by areas of residence. According to our results, adenocarcinoma was the predominant histological type in nearly all provinces.

**Table 2:** Frequency of colorectal cancer in Iran, by histological features and province, 2014–2017

**Province**	**Adenocarcinoma ** **No. (%)**	**Cystic, Mucinous and Serous Cancers No. (%)**	**Other Pathologies ** **No. (%)**	**Total** **Number**
Ardabil	543 (70.16)	41 (5.3)	190 (24.55)	774
Alborz	1032 (70.25)	124 (8.44)	313 (21.31)	1469
Isfahan	2563 (71.51)	290 (8.09)	731 (20.40)	3584
Ilam	326 (84.17)	11 (2.94)	37 (9.89)	374
East Azarbayjan	2043 (72.94)	134 (4.78)	624 (22.28)	2801
West Azarbayjan	1114 (69.84)	75 (4.70)	406 (25.45)	1595
Busher	197 (58.46)	15 (4.45)	125 (37.09)	337
Tehran	8267 (78.40)	976 (9.26)	1301(12.34)	10544
Chaharmahal Bakhteiari	286 (74.87)	18 (4.71)	78 (20.42)	382
Razavi Khorasan	2667 (73.15)	239 (6.56)	740 (20.30)	3646
Khoozestan	1257 (70.86)	196 (11.05)	321 (18.09)	1774
Zanjan	321 (74.13)	34 (7.85)	78 (18.01)	433
Semnan	330 (74.16)	33 (7.42)	82 (18.43)	445
Sistan Baloochestan	250 (66.14)	27 (7.14)	101 (26.72)	378
Fars	1400 (68.26)	169 (8.24)	482 (23.50)	2051
Ghazvin	472 (72.39)	32 (4.91)	148 (22.70)	652
Qom	361 (61.92)	25 (4.29)	197 (33.79)	583
Kordestan	384 (71.11)	33 (6.11)	123 (22.78)	540
Kerman	691 (68.62)	74 (7.35)	242 (24.03)	1007
Kermanshah	684 (81.62)	39 (4.65)	115 (13.72)	838
Kohkilooye Boyerahmad	161 (79.31)	17 (8.37)	25 (12.32)	203
Golestan	727 (73.96)	46 (4.68)	210 (21.36)	983
Guilan	1469 (73.01)	205 (10.19)	338 (16.80)	2012
Lorestan	220 (42.15)	34 (6.51)	268 (51.34)	522
Mazandaran	1525 (67.03)	132 (5.80)	618 (27.16)	2275
Markazi	540 (66.42)	49 (6.03)	224 (27.55)	813
Hormozgan	206 (52.82)	33 (8.46)	151 (38.72)	390
Hamedan	535 (63.84)	46 (5.49)	257 (30.67)	838
Yazd	529 (71.11)	65 (8.74)	150 (20.16)	744
South Khorasan	234 (74.52)	12 (3.82)	68 (21.66)	314
North Khorasan	172 (61.65)	18 (6.45)	89 (31.90)	279

 According to the projections available in INPCR, both the number of CRC cases and the ASR are expected to steadily increase until 2020 and 2025, with the majority of cases occurring in men (Figure 4 and Table S4). Statistical analysis showed that the number of patients will rise to 13,096 in 2020 and 17,812 in 2025. In addition, the ASR is expected to increase to 15.9 in 2020 and 17.7 in 2025.

## Discussion

 CRC is recognized as one of the cancers with a significant global burden.^1^ According to both national and global statistics, the incidence of this disease is on the rise, and it is projected to increase by 60% for colon cancer and 71.5% for rectal cancer by 2035.^24^ Iran, as one of the largest nations in the Middle East, is home to a diverse range of cultural and ethnic groups. Utilizing the most comprehensive cancer registry in the country, the INPCR, we conducted an extensive analysis of CRC trends from 2014 to 2025. Our study also includes the assessment of gender-specific disease rates across all regions, an investigation into CRC incidence in every province of Iran, an examination of CRC occurrence across different age groups, and the identification of the most prevalent CRC subtype.

 In Iran, there were 43 580 new cases of CRC registered between 2014 and 2017. As expected, it was more prevalent in males who accounted for 55.96% of the cases. Similarly, Lieberman et al reported that the incidence rate of CRC is 30% higher in men, with an even greater disparity observed for rectal cancer.^25^ Another study examining the incidence rates of CRC from 2000 to 2014 in the United States found that this rate was significantly higher among men compared to women.^26^ The difference in CRC rates between men and women can be attributed to several biological and behavioral factors. For example, men tend to have a higher propensity for smoking,^27^ consume larger quantities of alcohol,^28^ and follow diets rich in red and processed meat.^29^ Additionally, men are more likely to accumulate visceral fat,^30^ which is, in turn, linked to a higher chance of developing CRC.^31-33^

 We observed an increasing trend in the incidence rates of CRC in the Iranian population during 2014-2017, and this trend is projected to continue to rise until 2025. Iran is classified as a developing country in the world and holds the status of an upper-middle-income economy according to the World Bank. In a global evaluation of CRC incidence across 39 countries with varying Human Development Index (HDI) rankings, it has been noted that countries with medium to high HDI levels have shown an increasing trend in CRC occurrences in recent years, while high-HDI nations exhibit a decreasing trend.^34^ Another study by Xi and Xu in 2021 emphasized a growing trend of CRC in middle- and low-income countries. However, the authors argued that the incidence of CRC remains higher in well-developed countries.^35^ The increased burden of CRC in developing countries can be attributed to the adoption of a Western lifestyle.^11^ Over the past 50 years, dietary habits in these countries have shifted towards increased consumption of fats, sugar, and animal-source foods.^36^ Furthermore, economic progress has led to the development of sedentary jobs, resulting in reduced physical activity and subsequently higher rates of overweight and obesity, which are two major risk factors for CRC.^37^

 According to our findings, the incidence rate of CRC in both sexes significantly increases with the aging of the population, reaching a peak in the 80-84 age group (total ASR = 122.34). Ohri and colleagues obtained similar results when they evaluated CRC incidence by age among the American population. Based on their results, patients older than 80 years presented the highest incidence rate of CRC compared to other age groups.^26^ Similarly, an Iranian study reported the highest ASR for both sexes in the 80-84 age group.^38^ The direct association between increasing age and a higher incidence of CRC may result from the accumulation of various risk factors, such as genetic and epigenetic mutations over time.^39^ It is noteworthy that despite the majority of CRC cases occurring in older adults,^26,38^ its incidence trends are rising more significantly in people younger than 50 years.^5^ In a study by Siegel et al between 2000 and 2013, a 22% increase in the incidence rate of colon and rectal malignancies was documented in the under-fifty US population.^40^ Nevertheless, the increasing incidence of early-onset CRC is not limited to developed countries; it affects various regions, spanning from low-income countries to wealthy ones.^34^

 In this study, we also assessed the geographical distribution of CRC in Iran. Our findings revealed that both men and women living in Tehran had the highest ASR for CRC, while people from southeastern provinces such as Sistan-Balouchestan and Hormozgan had the lowest incidence of CRC. Our results align with a systematic review and meta-analysis of CRC in Iran, which concluded that the highest male ASR for CRC is reported in Tehran. However, the same result was not observed for women in Tehran.^41^ Another study conducted by Shadmani and colleagues explored CRC incidence based on geographical areas in Iran. Similarly, they discovered that the ASRs of CRC in Tehran ranked first among all provinces of Iran. Additionally, they reported the lowest incidence rates of CRC in the southeastern parts of the country, in accordance with our results.^38^

 Numerous studies have examined the factors contributing to geographical disparities in CRC. In addition to genetic determinants,^41^ most of these factors are associated with the socioeconomic status (SES) of the inhabitants, showing a controversial correlation with CRC incidence in different regions around the world.^42-45^ Some investigations reported a reverse association between the incidence of CRC and SES, similar to the present study.^42,43^ In other words, individuals living in deprived regions exhibited lower incidence rates of CRC compared to those residing in more affluent areas.^42,43^ Several factors may help explain these trends. First, the short-term increase in CRC incidence in areas with lower socioeconomic deprivation, such as Tehran in Iran, might be linked to enhanced screening programs.^46^ Second, unhealthy lifestyles and diets are more prevalent in higher socioeconomic regions, potentially contributing to an increased CRC incidence.^47^ Third, as many high socioeconomic areas are industrial regions, they may have elevated concentrations of potentially carcinogenic substances in the air, water, and soil.^48^ Regarding the lower incidence of CRC in southern Iran, one possible theory is that people in these regions have greater exposure to sunlight. Several studies suggest that vitamin D deficiency is a risk factor for developing CRC.^49-51^ Therefore, people living in the southern provinces may produce adequate levels of vitamin D, which could explain the lower incidence of CRC compared to the central and northern parts of the country.

 Adenocarcinoma is the most common subtype of CRC in Iran, a finding consistent with studies in other countries.^35,52,53^ Furthermore, according to global statistics, more than 90% of CRCs are adenocarcinomas derived from epithelial cells. Other types include neuroendocrine, squamous, adenosquamous, spindle cell, and undifferentiated types,^54^ which were not included in our study.

 Altogether, there has been a rising trend in CRC incidence in Iran over the past two decades.^55,56^ According to our results, the global incidence of this disease is expected to increase in the coming decade. Potential factors contributing to this trend include population aging, physical inactivity, obesity, and a diet high in protein and low in fiber. Additionally, improvements in screening systems and earlier patient detection can play a crucial role in the short-term increase in CRC incidence.

 The most significant advantage of this study is the utilization of the INCRS database, which covered 86.7% of the Iranian population. However, it is worth noting that this registry system did not include data from all cities and rural areas across the country. Additionally, we were unable to report tumor grades and anatomical details due to the absence of this data in the INPCR. Nevertheless, it is important to emphasize that these limitations did not impact the primary objective of our study, which was to report on CRC incidence.

## Conclusion

 In this study, we have reported the epidemiological features of CRC in the Iranian population between the years 2014 and 2017. The rising trend in the incidence of the disease necessitates further research to identify the underlying causes and risk factors. Moreover, there is a need to develop screening programs for the early diagnosis of patients to reduce the burden of CRC within the Iranian community. High-risk populations, including older adults (above 50 years), men, and residents of provinces with higher incidence rates, may benefit the most from these screening programs.

## Supplementary Files

Supplementary file 1 contains Figure S1 and Tables S1-S4.
