# Supplementary material for: Colorectal Cancer Incidence in Iran Based on Sex, Age, and Geographical Regions: A Study of 2014–2017 and Projected Rates to 2025
Source: Arch Iran Med. 2024 Apr 1;27(4):174–82. doi: 10.34172/aim.2024.26 (PMC11097305; doi:10.34172/aim.2024.26)
Supplement: Supplementary file 1 — contains Figure S1 and Tables S1-S4. [file aim-27-174-s001.pdf]

Arch Iran Med. April 2024;27(4):174-S1

10.34172/aim.2024.26

Supplementary file 1

Table S1. Age-standardized Incidence Rates (ASR) (per 100,000 person-year) of Colorectal Cancer in Iran by Province, 2014–2017.

**Province**	**2014**	**2015**	**2016**	**2017**
Ardabil	12.17	14.66	19.74	17.28
Alborz	11.96	11.78	16.45	17.78
Isfahan	15.83	16.39	16.08	18.02
Ilam	13.76	18.10	20.8	19.36
East Azarbaijan	17.75	14.08	17.51	17.98
West Azarbaijan	13.62	12.44	13.5	14.67
Busher	8.11	10.91	8.24	11.98
Tehran	18.9	14.79	21.61	20.46
Chaharmahal Bakhteiari	10.97	12.44	10.21	13.31
Razavi Khorasan	17.05	14.41	16.05	17.14
Khoozestan	12.43	11.15	11.19	13.20
Zanjan	12.26	9.42	10.65	10.62
Semnan	19.5	16.28	13.8	15.12
Sistan Baloochestan	6.91	5.33	6.2	5.66
Fars	10.07	12.27	8.97	13.01
Ghazvin	12.47	13.56	14.05	14.59
Qom	Not Available	17.98	18.39	18.43
Kordestan	8.17	7.86	9.44	10.29
Kerman	9.61	10.15	9.59	10.54
Kermanshah	10.2	9.51	10.81	11.85
Kohkilooye Boyerahmad	8.02	10.23	8.5	10.43
Golestan	15.74	14.41	15.88	17.22
Guilan	12.05	15.23	15.16	19.31
Lorestan	7.41	8.67	6.75	8.76
Mazandaran	12.02	15.13	15.2	17.95
Markazi	9.59	12.84	14.78	15.39
Hormozgan	6.34	8.94	6.97	8.79
Hamedan	10.4	10.77	11.24	12.20
Yazd	17.33	14.52	19.61	19.22
South Khorasan	13.33	9.29	11.5	9.77
North Khorasan	9.68	6.76	9.29	10.92

Table S2. Number and Age-standardized Incidence Rates (ASR) (Per 100,000 Person-Year) of Colorectal Cancer in Iran by Province, 2014–2017.

	Total		Male		Female	
**Province**	**Number**	**ASR**	**Number**	**ASR**	**Number**	**ASR**
Ardabil	774	16.03	425	17.93	349	14.28
Alborz	1469	14.64	865	17.37	604	11.89
Isfahan	3584	16.60	2043	18.85	1541	14.38
Ilam	374	18.06	188	17.61	186	18.43
East Azarbaijan	2801	16.83	1603	19.49	1198	14.24
West Azarbaijan	1595	13.57	869	15.11	726	12.11
Bushehr	337	9.88	196	11.60	141	8.34
Tehran	10544	18.99	5997	21.69	4547	16.33
Chaharmahal Bakhtiyari	382	11.74	213	13.48	169	10.12
Razavi Khorasan	3646	16.15	2049	18.33	1597	14.06
Khoozestan	1774	12.01	1018	13.98	756	10.11
Zanjan	433	10.72	225	11.47	208	10.10
Semnan	445	16.11	258	18.73	187	13.51
Sistan Baloochestan	378	6.01	188	5.99	190	6.06
Fars	2051	11.08	1128	12.30	923	9.91
Ghazvin	652	13.68	384	16.24	268	11.17
Qom	583	18.26	324	20.36	259	16.27
Kordestan	540	8.97	301	10.11	239	7.85
Kerman	1007	9.98	536	10.73	471	9.26
Kermanshah	838	10.64	457	11.62	381	9.64
Kohkilooye.Boyerahmad	203	9.35	108	9.78	95	8.92
Golestan	983	15.85	539	17.89	444	13.93
Guilan	2012	15.51	1146	18.12	866	13.03
Lorestan	522	7.90	290	8.98	232	6.93
Mazandaran	2275	15.15	1251	16.77	1024	13.56
Markazi	813	13.24	412	13.68	401	12.89
Hormozgan	390	7.80	209	8.41	181	7.21
Hamedan	838	11.16	456	12.37	382	10.05
Yazd	744	17.72	411	19.73	333	15.83
South Khorasan	314	10.95	165	11.70	149	10.23
North Khorasan	279	9.17	137	9.66	142	8.76

Table S3. Age-standardized Incidence Rates (Per 100,000 Person-year) of Colorectal Cancer in Iran, 2014–2017.

**Age group (years)**	**Total**	**Male**	**Female**
00_04	0.05	0.04	0.05
05_09	0.01	0.02	0.01
10_14	0.09	0.09	0.09
15_19	0.39	0.39	0.4
20_24	0.87	0.94	0.8
25_29	1.89	1.92	1.86
30_34	3.28	3.51	3.06
35_39	5.66	5.91	5.39
40_44	9.84	9.9	9.78
45_49	16.05	16.28	15.81
50_54	28.07	29.54	26.58
55_59	41.41	45.18	37.67
60_64	58.67	67.08	50.57
65_69	79.42	95.44	65.04
70_74	99.2	118.57	80.85
75_79	115.94	137.17	93.78
80_84	122.34	140.1	102.48
>=85	115.18	134.45	94.85

Table S4. Predicted Number and Age-standardized Incidence Rates (ASRs) (Per 100,000 Person-year) of Colorectal Cancers in Iran, 2020 and 2025.

	**Both male and female**		**Male**		**Female**	
**Year**	**Number**	**ASR**	**Number**	**ASR**	**Number**	**ASR**
**2020**	13096	15.9	7511	18.3	5830	14.0
**2025**	17812	17.7	10163	20.5	7989	15.8

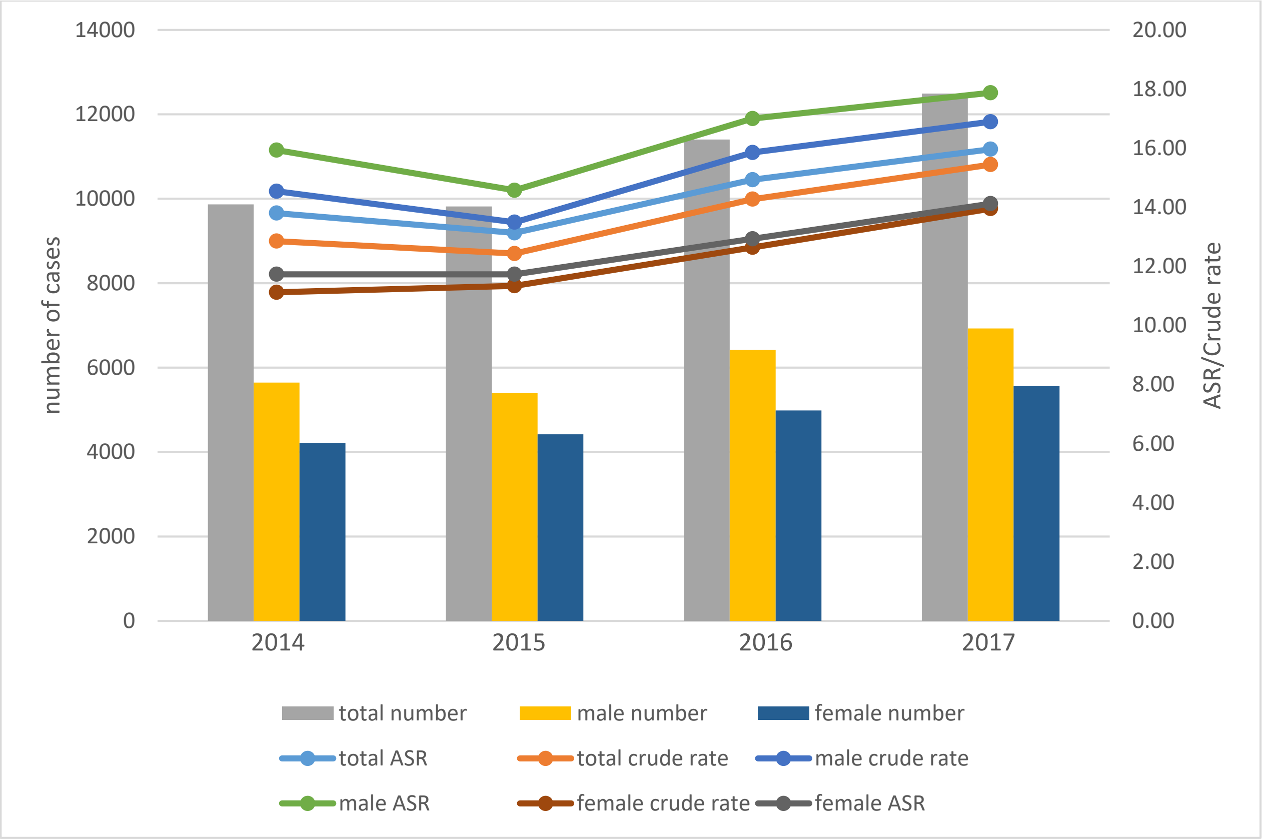

Figure S1. Number, crude rate, and age-standardized incidence rate (ASR) (per 100,000 person-year) of colorectal cancers in Iran, 2014–2017.
